# Bactericidal Antibody Responses to Meningococcal Recombinant Outer Membrane Proteins

**DOI:** 10.4014/jmb.2401.01018

**Published:** 2024-04-24

**Authors:** Ming Zhu, Yunqing Sun

**Affiliations:** Department of Pediatrics, Shandong Provincial Third Hospital, Shandong University, No.11 Wuyingshan, Middle Road, Tianqiao District, Jinan, Shandong 250031, P.R. China

**Keywords:** Bactericidal antibody, *Neisseria meningitidis*, PilQ_406-770_, rPorA

## Abstract

Secretin PilQ is an antigenically conserved outer membrane protein that is present in most meningococci and PorA is a major protein that elicits bactericidal immune response in humans following natural disease and immunization. In the present study, BALB/c mice were immunized subcutaneously with rPilQ406-770 or rPorA together with Freund’s adjuvant (FA). Serum antibody responses to serogroup A and B *Neisseria meningitides* whole cells or purified proteins and functional activity of antibodies were determined by ELISA and serum bactericidal assay (SBA), respectively. Serum IgG responses were significantly increased in the immunized group with rPilQ406-770 or rPorA together with FA compared to control groups. IgG antibody response of mice immunized with rPilQ406-770 was significantly more than mice immunized with rPorA (OD at 450 nm was 1.6 versus 0.83). The booster injections were effective in increasing the responses of anti-rPilQ406-770 or anti-rPorA IgG significantly. Antisera produced against rPilQ406-770 or rPorA demonstrated strong surface reactivity to serogroup B *N. meningitides* in comparison with control groups. Antisera raised against rPorA or rPilQ406-770 and FA demonstrated SBA titers from 1/1024 to 1/2048 against serogroup B. The strongest bactericidal activity was detected in sera from mice immunized with rPilQ406-770 mixed with FA. These results suggest that rPilQ406-770 is a potential vaccine candidate for serogroup B *N. meningitidis*.

## Introduction

*Neisseria meningitidis* causes half a million cases of septicemia and meningitis globally each year and remains an important public health problem [1]. While polysaccharide conjugate vaccines are available against infections caused by *N. meningitidis* serogroups A, C, Y and W135, there are no vaccines to prevent disease caused by strains of serogroup B, because cross-reactivity of the serogroup B capsule with surface glycoproteins of human fetal cells has hampered efforts to develop a reliable vaccine [2, 3]. This has prompted the evaluation of a number of noncapsular antigens, but none of these have yet provided broad protection against meningococci commonly associated with disease [4]. Obviously, identification of a protein that is present in all strains of serogroup B and elicits broader cross-protection against multiple serosubtypes is a highly desirable goal for serogroup B vaccine development [5, 6]. One of these proteins is the antigenically conserved outer membrane secretin PilQ that plays an essential role in the biogenesis of type IV pili and mediating pilus translocation across the outer membrane [7, 8]. Type IV pili are most important in the early stages of infection and demonstrated that Neisserial PilQ-null mutants are not piliated [9, 10]. PilQ is a member of the GspD secretin superfamily and is unique among them because of its abundance in the outer membrane and the presence in its N-terminal domain of four to seven copies of an octapeptide, PAKQQAAA, termed small basic repeats [11]. The C terminal of protein which is conserved between members of secretin superfamily and is responsible for secretin oligomerization has been predicted to contain 13 β-strands, which could be embedded in the outer membrane [7].

PilQ is an attractive vaccine candidate because it has a relatively conserved sequence, it is present on most meningococci and it is abundant on the cell surface. Wilde and colleagues demonstrated that antibodies raised to the PilQ multimer had bactericidal activities [11]. Recent studies have shown that outer membrane vesicles (OMVs) produced from a strain in which the *pilQ* gene was up regulated induced higher anti-PilQ antibody titers in mice [12].

Other important protein that has been shown to induce bactericidal immune responses in human serum following natural disease and immunization is PorA [13]. PorA as a class 1 outer membrane protein (OMP) is a major protein of 45 kDa that elicits a protective immune response in humans than for any other meningococcal surface proteins [14]. Recombinant OMV-formulations with various PorA antigens have been developed in some countries; therefore, it can be considered a reliable candidate antigen as a part of multi-component recombinant protein vaccine [15].

In the present study, the full coding sequence of PorA chosen and immunological evaluations were done. According to PilQ topology determined by Frye SA *et al*., particular region (406-770) is part of C terminal of protein which has a conserved sequence between members of secretin superfamily and exposed at the outer surface of outer membrane [11]. Because of these reasons, we were chosen this fragment of PilQ and evaluate of vaccine potential using active immunization and comparing it with recombinant PorA.

## Materials and Methods

### Bacterial Strains and Vector

*N. meningitidis* serogroup B (ATCC13090) and serogroup A (ATCC13077) were cultured on chocolate agar. *Escherichia coli* DH5α (Invitrogen, USA), BL-21(DE3) pLysS (Novagen, USA) and Origami B (DE3) (Novagen) were used for cloning and expression. pET-28a and pET-32a (Novagen) were used as an expression vector.

### Antigens and Adjuvants

A 1,095 bp fragment of C-terminal coding sequence of *pilQ* (pilQ_406-770_) and the full coding sequence of *porA* (1,200 bp) were amplified by PCR on chromosomal DNA of *N. meningitidis* serogroup B strain ATCC13090 and cloned into the pET28a and pET32a vectors, respectively. Recombinant plasmids were transformed into *E. coli* BL21 (DE3) and Origami B (DE3) to express PilQ_406-770_ and PorA as COOH terminal histidine fusion proteins, respectively. Protein expression was induced at 37°C by adding 1 mM isopropyl-β-D-thiogalactopyranoside (IPTG) at an optical density at 650 nm (OD650) of 0.7 and growing the bacteria for an additional 4 h. Expression was evaluated by sodium dodecyl sulfate polyacrylamide gel electrophoresis. PilQ_406-770_ -His and PorA -His fusion proteins were produced as insoluble inclusion bodies and were solubilized with urea and renatured after purification. The solubilized proteins were purified by affinity chromatography on a nickel-nitrilotriacetic acid (Ni-NTA) gel matrix (Qiagen, UK) under denaturing conditions. The purified proteins were dialyzed against 20 mM Tris– HCl, pH 7.4 for removing imidazole. The protein concentration was determined by Bradford assay with bovine serum albumin as standard and NanoDrop spectrophotometry (Bio-Rad, USA). Complete Freund's Adjuvant for the first dose and incomplete Freund's adjuvant for the second and third booster doses were used as an adjuvant [16, 17].

### Mouse Immunization

Groups of five-week-old BALB/c female mice were immunized subcutaneously (SC) with rPilQ_406-770_ (10 μg/ 50 μl) or rPorA (10 μg/ 50 μl) together with an equal volume (50 μl) of Freund's adjuvant (the final injection volume for each mouse was 100 μl). Mice were immunized at weeks 0, 2 and 4. Control groups consisted of mice receiving Freund’s adjuvant (FA) or phosphate buffered saline (PBS) alone. Sera were collected at weeks 0, 2, 4, and 6 to determine the antibody responses and functional activities. Aliquots of serum were stored at -70°C for assay.

### Determination of Serum IgG Levels to *N. meningitidis* Whole Cells and Purified Proteins

The IgG antibody responses to *N. meningitidis* serogroup A (ATCC13077) and serogroup B (ATCC13090) whole cells were determined by whole-cell ELISA on pooled sera collected at week 6 as previously described. Also, the IgG antibody responses against rPorA and rPilQ_406-770_ were determined by ELISA of individual serum samples collected at weeks 2, 4 and 6.

The checkerboard titration assay was used to determine the optimum concentration of each protein and the appropriate dilution of sera. The protein concentrations used in the checkerboard were 0.25, 0.5, 1 and 2 μg/100 μl and serum dilutions were 1:100, 1:200, 1:500 and 1:1000. So, the optimum concentration of each protein was 1 μg/ 100 μl and optimum dilution of sera was 1:500. Flat-bottom, 96-well microtiter plates (Immulon 2B; Thermo Electron Corp.) were coated overnight at 37°C with 1 μg/100 μl of proteins in PBS. The coated plates were first blocked with 5% (wt/vol) nonfat milk in PBS and then incubated with antisera (1:500 diluted in PBS/BSA) for 2 h at 37°C. Plates were washed three times with PBS-T and rabbit anti-mouse immunoglobulin G (IgG)–peroxidase conjugate diluted 1:7000 in PBS added. Plates were incubated for 1 h at 37°C and washed three times in PBS-T, then 3, 3', 5, 5'-Tetramethylbenzidine (TMB) substrate added to each well. Reaction was stopped after 30 min by adding of H_2_SO_4_ to 2N and the absorbance was measured at 450 nm. All assays were performed in triplicate.

### Serum Bactericidal Assay

The bactericidal assay was carried out using strains ATCC13090 and ATCC13077 as described previously [18]. 10% (v/v) baby rabbit complement was used as an exogenous complement source and bactericidal assay was performed on pooled serum specimens collected at weeks 0 and 6 and all sera were heat inactivated for 30 min in 56°C [19-22]. Sterile 96-well flat-bottom plates were used for serum bactericidal assay (SBA). The mixture contained 25 μl of serially diluted sera in Dulbecco’s buffer, 12.5 μl of Dulbecco’s buffer containing 300 colony forming unit (CFU) of bacteria, and 12.5 μl of complement (20% vol/vol). After all of the components were added to each well, a 10 μl aliquot of each well was spotted onto a chocolate agar plate and incubated overnight at 37°C in a 5% CO_2_ atmosphere. 96-well plates were incubated for 90 min at 37°C and then, a 10 μl aliquot was taken from each well and spotted onto a chocolate agar plates. The percentages of bacteria surviving were calculated by comparing the respective CFU at 90 min with that at time zero in negative control samples. Bactericidal titers were reported as the reciprocal of the highest dilution of test serum that yielded ≥ 50% bacterial killing compared to assay controls. Specimens that demonstrated ≤ 50% killing at the lowest serum dilution tested (the lowest dilution tested for serum samples was 1:25) were reported as having a SBA titer of ≤ 25. All assays were performed in duplicate.

### Statistical Analysis

All the statistical analysis was done by using SPSS statistical software package (version 22.0). Following normalization of the data, one-way ANOVA (LSD) and repeated measures tests were used to check for differences between data sets and Bartlett’s test was used to determine variance within data sets. The optical density of ELISA was expressed as log_10_ value of the geometric mean obtained for each group of mice. *P* values ≤0.05 were considered statistically to be significant.

## Results and Discussion

### Antigens Preparation

**Fig. 1:**
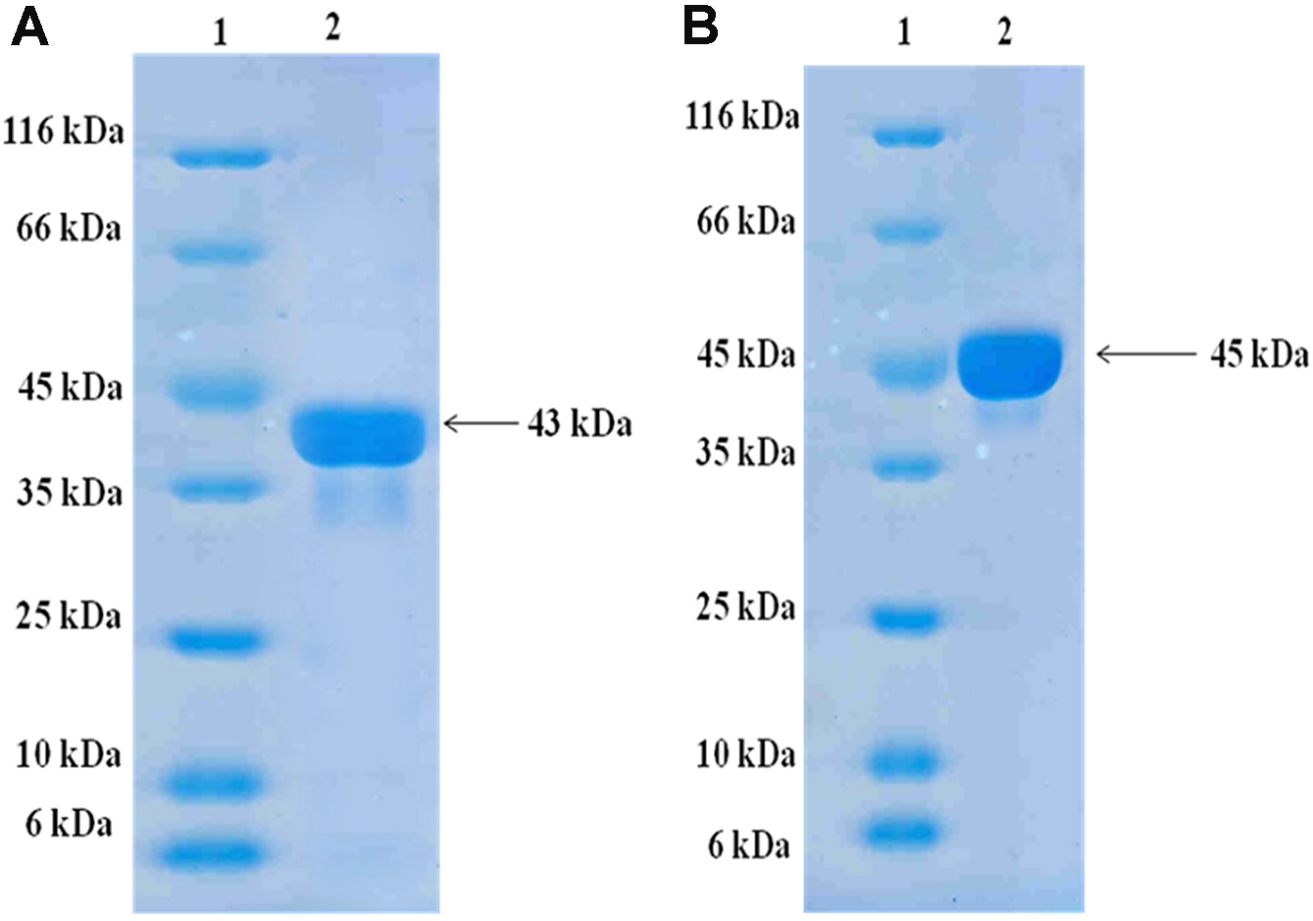
SDS-PAGE (12% w/v) of purified recombinant PilQ_406-770_ and PorA stained with Coomassie-blue G-250. (**A**) Lane1: Standard protein size marker, lane 2: Purified recombinant PilQ_406-770_ from Ni^2+ -^Sepharose column with molecular weight of 43 kDa. (**B**) Lane1: Standard protein size marker, lane 2: Purified recombinant PorA from Ni^2+ -^Sepharose column with molecular weight of 45 kDa.

The 406-770 particular region of PilQ C-terminal (43 kDa) and the full lenght of PorA protein (45 kDa) were chosen and expressed into *E. coli* BL21 (DE3) and Origami B (DE3), respectively. Recombinant proteins in the form of dissoluble inclusion bodies were carefully purified with Ni-NTA affinity chromatography under denaturing condition (Fig. 1). The refolded, affinity-purified proteins preparation contained ≥ 95% recombinant proteins together with some minor contaminants from the *E. coli* expression host. In order to check the lipopolysaccharide (LPS) level in the produced and purified recombinant proteins, LAL assay (Sigma-Aldrich) was used. The results revealed that the amount of LPS present in the samples was very small and less than 1 EU/ml, which is acceptable for animal injection.

### Serum IgG Responses after SC Immunization

**Fig. 2:**
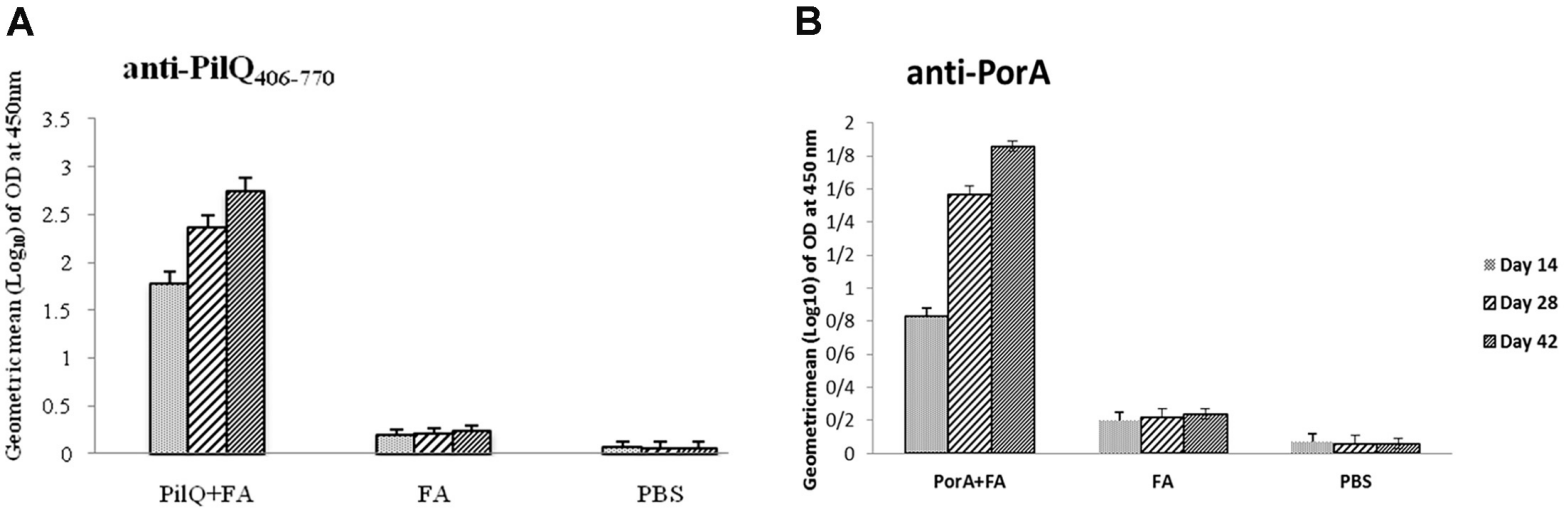
IgG antibody responses against PilQ_406-770_ or PorA measured by ELISA of individual serum samples collected at weeks 2, 4 and 6. The bars represent the Geometric mean (log10) of obtained ELISA signals for optimum dilution (1:500). Optimum dilution was determined prior to the comparisons, by testing serially diluted sera against the coated antigen. Error bars indicate ±SE in each group. IgG antibody responses of mice immunized with PilQ_406-770_ was higher than mice immunized with PorA (*P* ≤ 0.001). The booster injections were effective to significantly increase the responses of anti-PilQ_406-770_ or anti-PorA IgG (*P* ≤ 0.001). All assays were performed in triplicate.

**Fig. 3:**
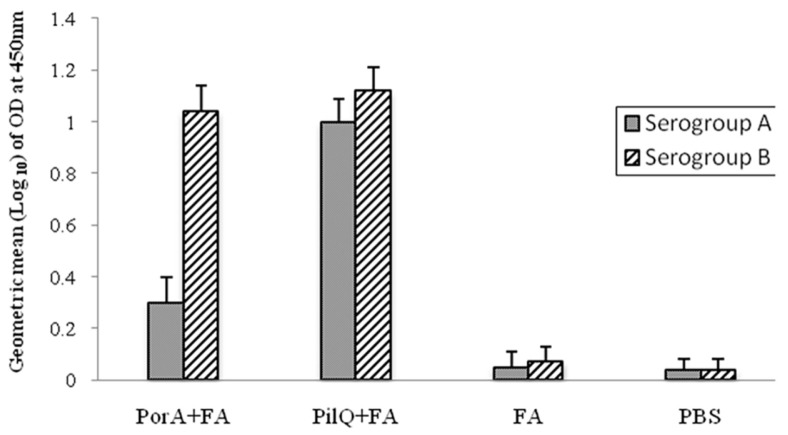
IgG antibody responses against heat killed *N. meningitidis* serogroups A and B were determined by Whole-cell ELISA on pooled sera collected at week 6. The bars represent the Geometric mean (log10) of obtained ELISA signals for optimum dilution (1:500). Optimum dilution was determined prior to the comparisons, by testing serially diluted sera against the coated antigen. Error bars indicate ±SE in each group. Antisera produced against rPilQ406-770 or rPorA demonstrated strong surface reactivity to serogroup B *N. meningitides* in comparison with control groups (*P* ≤ 0.001). All assays were performed in triplicate.

BALB/c mice immunized SC with rPilQ_406-770_ or rPorA together with Freund’s adjuvant exhibited high levels of specific antibody responses in comparison with control groups (*P* ≤ 0.001) (Fig. 2). IgG antibody response of mice immunized with rPilQ_406-770_ was significantly more than mice immunized with rPorA (*P* ≤ 0.001). The booster injections, especially the second booster, were effective to significantly increase the responses of anti-rPilQ_406-770_ or anti-rPorA IgG (*P* ≤ 0.001). The antisera were tested in whole-cell ELISA against native PorA and PilQ proteins present in the OM of strains ATCC13077 and ATCC1390. Antisera produced against rPilQ_406-770_ or rPorA demonstrated strong surface reactivity to serogroup B *N. meningitides* in comparison with control groups (11 folds higher) (*P* ≤ 0.001) (Fig. 3). Although, antisera surface reactivity to serogroup B from mice immunized with rPilQ_406-770_ was higher than mice immunized with rPorA, but these difference wasn’t significant (*P* > 0.05). Furthermore, rPilQ_406-770_ antisera demonstrated strong surface reactivity to serogroup A *N. meningitides*, but rPorA antisera didn’t show. Surface reactivity to serogroup B *N. meningitidis* from mice immunized with rPilQ_406-770_ was higher than serogroup A.

### Serum Bactericidal Assay

Bactericidal antibodies are an important correlate of protection against meningococcal infection. The sera from rPilQ_406-770_ or rPorA immunized animals were strongly bactericidal against serogroup B in comparison with control group (7-8 folds higher). Antisera raised against rPorA or rPilQ_406-770_ together with FA demonstrated SBA titers from 1/1024 to 1/2048 against serogroup B. The strongest bactericidal activity was detected in sera from mice immunized with rPilQ_406-770_ mixed with FA. The antisera were also tested for their ability to kill the heterologous serogroup A [23-25]. Although, antisera raised against the rPilQ_406-770_ showed high levels of bactericidal activity towards the heterologus strain, but rPorA antisera didn’t show bactericidal activity against heterologus strain (Table 1).

The outer membrane proteins in Gram negative bacteria have particular significance as a potential target for protective immunity. Meningococcal PilQ is an antigenically conserved, abundant outer membrane protein which forms a large multimer composed of 10 to 12 subunit and is a key component of the type IV pilus secretion machinery [26]. A total of 200 to 300 conserved C-terminal residues of PilQ exhibits identity with members of GspD superfamily [27]. Previous studies have shown that PilQ is immunogenic and is also a target for bactericidal antibodies following immunization [28]. Halliwell *et al*. demonstrated that immunization with PilQ complex protected mice against experimental infection. Recent studies have shown that OMVs produced from a strain in which the pilQ gene was up regulated induced higher anti-PilQ antibody titers in mice [12].

Meningococcal class 1 outer membrane protein (PorA) is a major component of the outer membrane and functions as a cationic porin [29]. Previous studies have shown that PorA is immunogenic during natural infection and is also a target for bactericidal antibodies, following immunization with experimental OMVs [30, 31]. In clinical vaccination trials with OMVs, it has been shown that PorA is critical for the induction of bactericidal antibodies in humans [32]. The main difficulty with PorA antigens is that they are antigenically diverse and display considerable temporal and geographical variability [33].

In the present study, we constructed a recombinant fragment of PilQ protein from residues 406 to 770 termed as PilQ_406-770_ and evaluate of vaccine potential using active immunization and comparing it with recombinant PorA. According to results, Anti-rPilQ_406-770_ IgG responses were significantly increased in group immunized with rPilQ_406-770_ in comparison with rPorA immunized group. Antisera from mice immunized with rPilQ_406-770_ demonstrated surface reactivity to serogroup A and B, but rPorA antisera showed only surface reactivity to serogroup B. The strongest bactericidal activity against serogroup A and B was detected in sera from mice immunized with rPilQ_406-770_. We demonstrate that utility of purified rPilQ_406-770_ together with Freund’s adjuvant elicits antibodies capable of inducing bactericidal activity against serogroups A and B *N. meningitidis*. Although, antisera raised against the rPilQ_406-770_ showed high levels of bactericidal activity towards the heterologus strain, but rPorA antisera didn’t show bactericidal activity against heterologus strain.

## Conclusion

The capacity of rPilQ_406-770_ to induce systemic bactericidal antibodies against homologus and heterologus strains, together with its relatively conserved sequence and abundance on the cell surface, makes it a potential candidate for inclusion in a vaccine against serogroup B meningococci.

## Figures and Tables

**Table 1:** Serum bactericidal activity elicited by vaccine candidates.

Bactericidal titer (BC50 titer^a^)				
Test strain	rPilQ_406-770_+FA	rPorA+FA	FA	PBS
Serogroup A (ATCC13077)	* 1024	≤ 8	≤ 8	≤ 8
Serogroup B (ATCC13090)	*2048	*1024	≤ 8	≤ 8

*P values ≤0.05

^a^The bactericidal (BC50) titers are expressed as the reciprocal of the greatest serum dilution that yielded ≥50% of bacterial killing, compared to assay controls. Bactericidal titers represent the GMT of the titers on pooled sera collected at weeks 0 and 6. All assays were performed in duplicate. Week 0 normal mouse sera had BC50 titers of ≤ 8.
